# Hyaluronan breakdown by snake venom hyaluronidases: From toxins delivery to immunopathology

**DOI:** 10.3389/fimmu.2023.1125899

**Published:** 2023-03-17

**Authors:** Felipe Silva de França, Denise V. Tambourgi

**Affiliations:** Immunochemistry Laboratory, Instituto Butantan, São Paulo, Brazil

**Keywords:** hyaluronidases, animal venoms, hyaluronan, high molecular weight hyaluronan, low molecular weight hyaluronan, damage associated molecular patterns, inflammation

## Abstract

Snake venom enzymes have a broad range of molecular targets in plasma, tissues, and cells, among which hyaluronan (HA) is outstanding. HA is encountered in the extracellular matrix of diverse tissues and in the bloodstream, and its different chemical configurations dictate the diverse morphophysiological processes in which it participates. Hyaluronidases are highlighted among the enzymes involved in HA metabolism. This enzyme has been detected along the phylogenetic tree, suggesting that hyaluronidases exert multiple biological effects on different organisms. Hyaluronidases have been described in tissues, blood and snake venoms. Snake venom hyaluronidases (SVHYA) contribute to tissue destruction in envenomations and are called spreading factors since their action potentiates venom toxin delivery. Interestingly, SVHYA are clustered in Enzyme Class 3.2.1.35 together with mammalian hyaluronidases (HYAL). Both HYAL and SVHYA of Class 3.2.1.35 act upon HA, generating low molecular weight HA fragments (LMW-HA). LMW-HA generated by HYAL becomes a damage-associated molecular pattern that is recognized by Toll-like receptors 2 and 4, triggering cell signaling cascades culminating in innate and adaptive immune responses that are characterized by lipid mediator generation, interleukin production, chemokine upregulation, dendritic cell activation and T cell proliferation. In this review, aspects of the structures and functions of HA and hyaluronidases in both snake venoms and mammals are presented, and their activities are compared. In addition, the potential immunopathological consequences of HA degradation products generated after snakebite envenoming and their use as adjuvant to enhance venom toxin immunogenicity for antivenom production as well as envenomation prognostic biomarker are also discussed.

## 1 Introduction

The diffusion of venom toxins from the inoculation site to the bloodstream is essential for the envenomation process/success. The degradation of extracellular matrix components (ECM), such as proteins and glycosaminoglycans (GAGs), is necessary for successful envenomation (1, 2). Together with other components of the ECM, GAGs produce a gel that is quite important for several morphophysiological processes, including maintenance of tissue stability and structure. These components present viscous characteristics, which delay microorganisms penetration into the tissues. Among the GAG targets of virulence factors from several pathogenic microorganism species and animal venom enzymes, hyaluronan (HA) has been highlighted (1, 3, 4).

HA is degraded by enzymes known as hyaluronidases (HYAs), which are present in venoms from snakes, lizards, and arthropods, as well in mammalian tissues and the bloodstream. Due to their functions in infections and envenomations, these enzymes are also called spreading factors (1, 5). Interestingly, the degradation products resulting from mammalian Hyaluronidases (HYAL) actions upon HA have been reported as inflammatory triggers, since they act as damage-associated molecular patterns (DAMPs) (3, 6–10), suggesting that hyaluronidases present in snake venoms (SVHYA) might be potentially inflammatory agents by generating HA fragments.

In this review, we summarize the structural and functional properties of HA and hyaluronidases from mammalian tissues and snake venoms. The similarity of the inflammatory and adjuvant properties of HA degradation products generated by HYAL and SVHYA are also compared.

## 2 Venomous snakes and venom composition

It has been estimated that 4,038 snake species exist in the world (11) from which around 15% present human and veterinary medical importance (12). The venomous snakes belong to the Colubroidea superfamily, and the most dangerous species are grouped in Viperidae (*e.g.*, true vipers and pit vipers) and Elapidae (*e.g.*, coral snakes, cobras, mambas) families (13). In addition, several species from the Colubridae (*e.g.*, boomslang and twig snakes) (14–16) and Lamprophiidae (*e.g.*, mole vipers and scaled burrowing asp) (17, 18) families are also considered of medical importance, since they can cause severe and fatal accidents.

The diversity of clinical symptoms observed in these accidents is associated with various factors as gender, age, body weight and genetic background of the victim (19–21), as well venoms composition (22, 23).

Snake venoms are complex chemical cocktails, which present a myriad of effects both in prey and human victims. These cocktails are composed by a plethora of components including ions, free amino acids, peptides, nucleotides, carbohydrates, lipids, biogenic amines, and proteins. Many of these molecules have as their main biological function immobilize or promote the death of the prey and aid in the digestion process (1, 23–25). Besides, venom composition diversity is responsible for the multitude of pathological effects observed in human victims (13).

Various classes of proteins can be found in the snake venoms as Hyaluronidases, Phospholipases A2, Snake Venom Metallo- and Serineproteases, and Three Finger Toxins, among others (1, 13). Although the presence of some venom components can vary depending on the species, genus or family of the snake, there is one that is ubiquitously encountered, the Hyaluronidase.

### 2.1 Hyaluronidases

HYAs production has been observed along the phylogenetic tree, from bacteriophages and other viruses, pathogenic bacteria, fungi, and invertebrates to vertebrate animals (26–28). In vertebrates, different cell types produce these enzymes, and they are detected in the ECM of diverse organs, including the testis, eyes, skin, spleen, liver, kidney, and uterus, and in secretions, including serum, semen and animal venoms (29) (**Table 1**).

**Table 1:** Comparison between HYAL and SVHYA.

Molecular characteristics	HYAL	SVHYA	Reference
Enzyme class	Hydrolases	Hydrolases	30
Enzyme subclass	Glycosidases	Glycosidases	30
Classification according to molecular reactions	EC 3.1.2.35	EC 3.1.2.35	26, 27, 30
Molecular weight range	7 – 320 kDa	33 – 110 kDa	29, 31–34
Presence of isoforms	+	+	29, 31–34
Substrates	Hyaluronan, Chondroitin sulfate A and C, Dermatan sulfate and β-heparin	Hyaluronan	29, 31–34
Optimal pH	3.3 – 7.0	3.0 - 7.0	29, 31–34
Residues involved with catalysis	Glu^149^, Asp^147^, Tyr^220^, Trp^341^	Glu^149^, Asp^147^, Tyr^220^, Trp^341^	2, 29
Residues involved with the HA specificity	Tyr^265^	Tyr^265^	2
Products generated after HA cleavage	Tetra- and Hexassacharides	Tetra- and Hexassacharides	29, 31
HA fragments generated by HYAs triggers inflammation	+	Must be investigated	3, 6–10
The Table was prepared with Word Processor Microsoft 365 (Office 365)			

HYAs enzymes, also called hyaluronoglucosaminidases, are members of the class of hydrolases, a subclass of glycosylases (EC 3.2). These enzymes function as glycosidases (EC 3.2.1) due to their ability to hydrolyze *O-* and *S-*glycosyl compounds (30). HYAs are glycoproteins with a broad range of molecular weights from 7 to 320 kDa. The optimal pH for their action can vary from 3.3 to 7.0 (29). According to the molecular substrates and products generated by HYAs enzymatic reactions, these enzymes are classified into three main subclasses (26–30):

1. HYAs (EC 3.1.2.35): This subclass includes hyaluronoglucosaminidases present in semen, serum, tissues, and lysosomes, as well as in hymenopteran and snake venoms. They possess transglycosidase and hydrolytic activities. Among the substrates of these enzymes, hyaluronan is highlighted. In addition, these enzymes act on chondroitin sulfate A and C and to a lesser extent on dermatan sulfate (chondroitin sulfate B) and β-heparin. The main product of their catalytic activity is the tetrasaccharide GlcUA-GlcNAc-GlcUA-GlcNAc.
2. HYAs (EC 3.1.2.36): This subclass includes hyaluronoglucuronidases that hydrolyze hyaluronan, resulting in the release of tetra- and hexasaccharides. These enzymes have been reported in leeches, parasites, and crustaceans.
3. HYAs (EC 4.2.2.21): This HYA group is produced by bacterial species and is characterized as HA lyases. They degrade HA, dermatan sulfate, and chondroitin sulfate A and C. These enzymes are called endo-β-N-acetyl-D-hexosaminidases, which act *via* β elimination since their catalytic activity generates disaccharides.

The molecular mechanisms of catalysis and substrate specificity are dictated by the presence of positional and structural catalytic residues conserved in the species in which HYAs were identified. The amino acid residues that characterize this enzymatic class are Glu^149^, which is important for the catalytic mechanism; the Asp^147^, Tyr^220^, Trp^341^ triad, which is responsible for positioning the carbonyl acetamide group for catalysis; and Tyr^265^, which is responsible for the HYAs specificity for HA. The replacement of the Tyr^265^ residue for Cys^265^ switches HYA specificity to chondroitin (2, 29).

### 2.2 Snake venom hyaluronidases

The initial data for the SVHYA were obtained during the 1930s. These studies showed that venoms contained a spreading factor that was able to increase tissue and blood capillary permeability to Indian ink and to pathogenic bacterial species. Some authors postulated that this factor would be important for venom absorption by prey and human victims (35, 36). In subsequent decades, the presence of spreading factors involved in efficient toxin delivery was ubiquitously detected in snake venoms. These factors, which include snake venom metalloproteinases and SVHYA, are important factors in tissue destruction since their actions are responsible for ECM breakdown (2, 29). SVHYA potentiate hemorrhaging, swelling, muscle damage and lethal effects of purified venom toxins, since its inhibition by monoclonal antibodies and plant derivative inhibitors substantially decreased the toxic effects of the venoms (1, 31–34). Thus, based on the available data, SVHYA are considered the main snake venom spreading factors.

Similar to HYAL, SVHYA are glycoproteins; however, their molecular weight ranges from 33 to 110 kDa, and they are generally produced as single chain polypeptides (29). In addition, more than one isoform has been reported in some venoms (1, 31). Harrison and colleagues (2) scrutinizing cDNA libraries and protein sequences showed that SVHYA conserve positional and structural catalytic residues that characterize this enzyme group.

Although hyaluronidases are ubiquitously expressed in snake venoms, the mechanisms involved in their effect on HA, which is present in the ECM and bloodstream, and the inflammatory consequences of these actions are underexplored. Biochemical studies examining the structure and activity of SVHYA clustered these enzymes in the EC 3.2.1.35 subclass together with HYAL (2, 33), which were previously shown to trigger inflammatory events (3, 6–10). Additionally, like HYAL, SVHYA act on HA to generate tetra- and hexasaccharides, suggesting that they potentially exert immunopathological effects.

## 3 HA and its hyaluronidase cleavage products

### 3.1 HA structure and function

**Figure 1:**
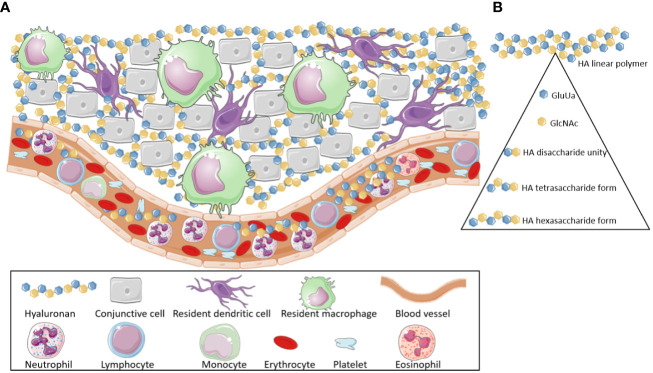
Hyaluronan location and composition. **(A)** Hyaluronan molecules are broadly found through the body, mainly in the extracellular matrix of different tissues. Additionally, they can also be detected in the bloodstream. **(B)** Hyaluronan is a high molecular weight polymer (>800 kDa). It is composed by subunits of D-glucuronic acid (GluUA) and N-acetyl-D-glucosamine (GlcNAc) that are attached, via glucuronic beta 1-3 linkage, thus forming disaccharide unities, which are the structural base of the Hyaluronan polymers. These disaccharide unities, when linked through hexosaminic beta 1-4 bonds (between GlcUA and GlcNAc), generate tetrasaccharides. The association of various tetrasaccharide units leads to the formation of the high molecular weight hyaluronan (HMW-HA). Low Molecular Weight Hyaluronan fragments (LMW-HA) are released, after cleavage of HMW-HA, by hyaluronidases and -hexosaminidases enzymes or by Reactive Oxygen Species. These fragments are Damage Associated Molecular Patterns (DAMPs), which act as pro-inflammatory and immunopathological agents. This Figure was partly generated using Servier Medical Art, provided by Servier, licensed under a Creative Commons Attribution 3.0 unported license. Of note, some icons in this figure were adapted for our necessities.

HA is a glycosaminoglycan that is mainly present in the vertebrate connective tissue ECM (**Figure 1A**) and has a broad range of morphophysiological functions. Structurally, HA is a large, linear, nonsulfated polymer characterized by repeating disaccharide units composed of D-glucuronic acid (GluUA) linked to N-acetyl-D-glucosamine (GlcNAc) (**Figure 1B**). These disaccharide units are attached by a glucuronic beta 1-3 linkage between GlcUA and GlcNAc and a hexosaminic beta 1-4 bond between GlcUA and GlcNAc (7–39). These disaccharide units are repeated a plethora of times assembling the HA high molecular weight form found in tissues (38) (**Figure 1A**).

HA is produced by a group of enzymes known as hyaluronan synthases (HAS). In mammalians, three HAS (*HAS1, HAS2, HAS3*) isoforms have been detected, which are expressed by different genes and participate of different steps of the HA biosynthesis (39).

Primary structure analysis showed that all HAS enzymes contain multiple clusters of hydrophobic amino acids at both the amino and carboxyl terminus, indicating that they are inserted into the lipid bilayer of the cellular membrane (39).

The HAS enzymes assemble HA polymers through Uridine diphosphate (UDP)-sugar precursors (UDP-GlcUA and UDP-GlcNAc), which are present at the cytoplasm. These enzymes contain a double catalytic domain, able to interact with two different substrates and generate disaccharide units necessary to the formation of structures that culminate with the generation of High Molecular Weight (HMW-HA) polymers. These molecules can remain associated to the cell membrane/HAS or to be extruded to the ECM (39).

HA is detected in different tissues with diverse biochemical characteristics and at different concentrations, which are crucial aspects that dictate the broad range of biological and pathological properties of this glycosaminoglycan (37–39).

Usually, HA is encountered in its native/homeostatic form as an HMW-HA linear polymer (**Figure 1B**). In this form, it has a molecular weight > 800 kDa (2, 37–40).

**Figure 2:**
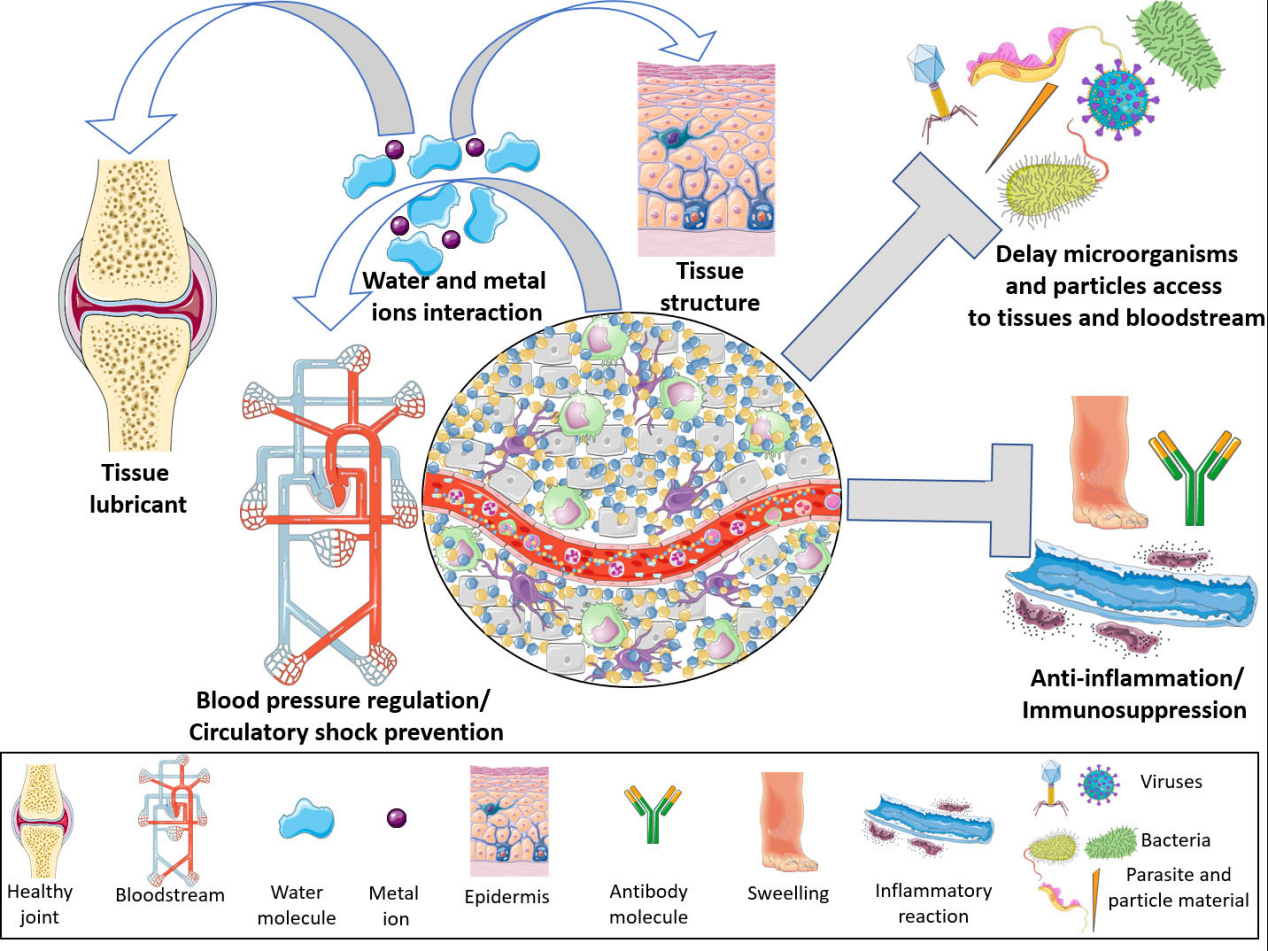
Hyaluronan physiological properties. On its high molecular weight form, hyaluronan is able to control a multitude of body functions. By interacting with high amounts of water and ions, hyaluronan becomes a potent regulator of the blood volume, thus controlling blood pressure and preventing a circulatory shock. Additionally, taking into account its chemical interactions, high molecular weight hyaluronan lubricates several tissues dampening physical friction, as well as contributing for the maintenance of tissues architecture. By interacting with water and extracellular matrix molecules, hyaluronan forms a mash which delays viruses, bacteria, parasites and material particles penetration into tissues and bloodstream. High molecular weight hyaluronan polymers are potent immunomodulators. These molecules abrogate innate and adaptive immune responses by blocking Toll Like Receptors or sequestrating cytokines and growth factors. This Figure was partly generated using Servier Medical Art, provided by Servier, licensed under a Creative Commons Attribution 3.0 unported license. Of note, some icons in this figure were adapted for our necessities.

In the HMW-HA form, this proteoglycan is highly hydrophilic, a characteristic that enables it to interact with several liters of water and metal ions; thus, it is a potent tissue lubricant (2, 37–39). In this form, it confers tissue stability and maintains the ECM architecture (3) by forming a network that, together with other components, delays microorganism infiltration into tissues (5). In addition, considering that HA is able to interact with high quantities of water, it is possible that these molecules could act as intravascular volume expander in life threatening extreme conditions to prevent circulatory collapse in several pathological disorders, such as septic shock (41) (**Figure 2**).

In addition to interactions with water, metallic ions and ECM components, HA activates diverse cell signaling cascades that elicit several biological responses, including metabolism, cell growth, proliferation, migration, mucus secretion and prosurvival pathways. Among the main receptors through which HA exerts its effects, CD44, HA-mediated motility receptor (RHAMM) and HA receptors for endocytosis (HARE; STAB2) have been identified. The signals triggered by these receptors culminate in the MAPK (mitogen-activated protein kinase), ERK1/2 (extracellular signal-regulated kinases 1/2) and NFκB (nuclear factor kappa B)-mediated gene expression pathways (37, 38, 42–47). Interestingly, the signaling pathways triggered by HA have been identified in recent decades as important for inflammatory reactions.

**Figure 3:**
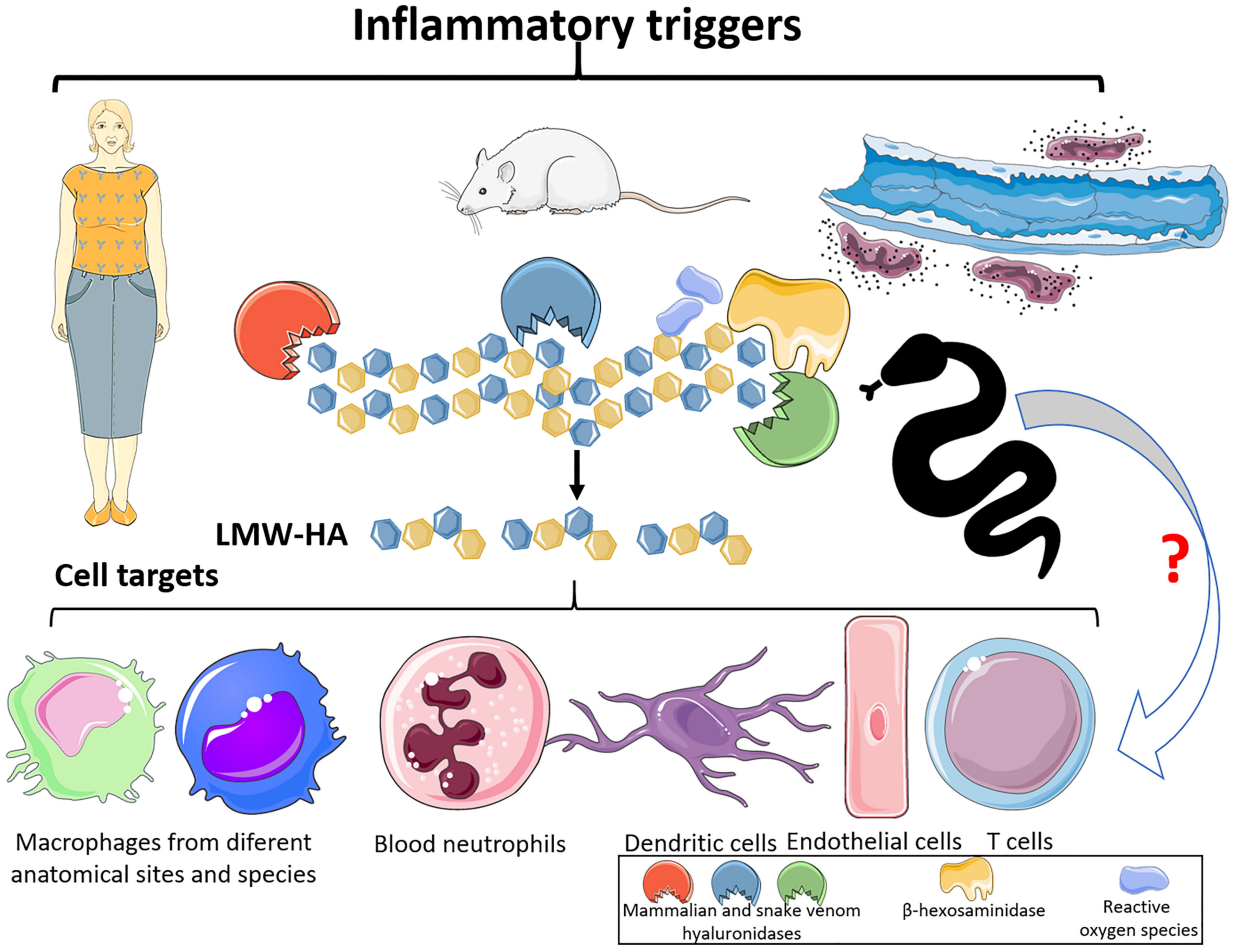
Hyaluronan fragmentation and its targets. After mammalian tissue and organ dysfunctions, elicited by diverse pathological conditions, hyaluronidases, b-hexosaminidases and ROS are released. These molecules can promote the cleavage of high molecular weight polymeric hyaluronan and generate tetra and hexasaccharide fragments (LMW-HA). These LMW-HA fragments function as DAMPs, which can be sensed by immune cells, via Toll Like Receptors -2 and -4. This event can lead to the production of interleukins, chemokines, and lipid mediators, leukocytes infiltration, as well as tissue damage. Hyaluronidases from snake venoms, as the mammalian molecules, can also act upon hyaluronan and release tetra and hexasaccharide fragments, which suggests that snake venom hyaluronidases can be potent inducers of inflammatory events via LMW-HA. This Figure was partly generated using Servier Medical Art, provided by Servier, licensed under a Creative Commons Attribution 3.0 unported license. Of note, some icons in this figure were adapted for our necessities.

Besides to the maintenance of tissue morphophysiological parameters, HA is considered an important immune surveillance molecule since it modulates a broad range of immunological reactions (3). In its physiological form, the HMW-HA polymer exerts anti-inflammatory/immunosuppressive (**Figure 2**) effects through diverse molecular mechanisms, including Toll-like receptor 2 (TLR-2) and TLR-4 blockade and cytokine and growth factor sequestration (3, 10, 48). However, in the sites of tissue injury and inflammation, HMW-HA polymers are degraded by HYAL and β-hexosaminidase enzymes and by reactive oxygen species (ROS) (3, 9), culminating in the generation of low molecular weight hyaluronan fragments (LMW- HA) (tetra- and hexasaccharide) (**Figure 3**) that are highly proinflammatory. Thus, LMW-HA becomes a damage-associated molecular pattern (DAMP), which serves as an indicator of endogenous injury/dysfunction, and is recognized by pattern recognition receptors (PRRs) (*e.g.*, TLRs, inflammasomes, and complement components) to trigger inflammation (3, 6–10).

### 3.2 Inflammatory effects of LMW-HA

Although HA-LMW can signal through the abovementioned receptors, the main signaling pathways involved in the inflammatory response to those fragments are mediated by TLR-2 and -4 activation and downstream molecules involved in this signaling cascade (*e.g.*, MyD88/PKC-ζ (protein kinase C ζ)/MAPK/ERK1-2/p38MAPK/p42-44/Scr/NFκB). These pathways are crucial for HA-induced inflammatory events, since knockout and mutation of these molecules and receptors in mice, as well as genetic interference and pharmacological modulation, abrogate these reactions (3, 6–10).

Macrophages from different anatomical sites and species, neutrophils, endothelial cells (ECs) and dendritic cells (DCs) can be highlighted as LMW-HA-responsive cells (**Figure 3**).

**Figure 4:**
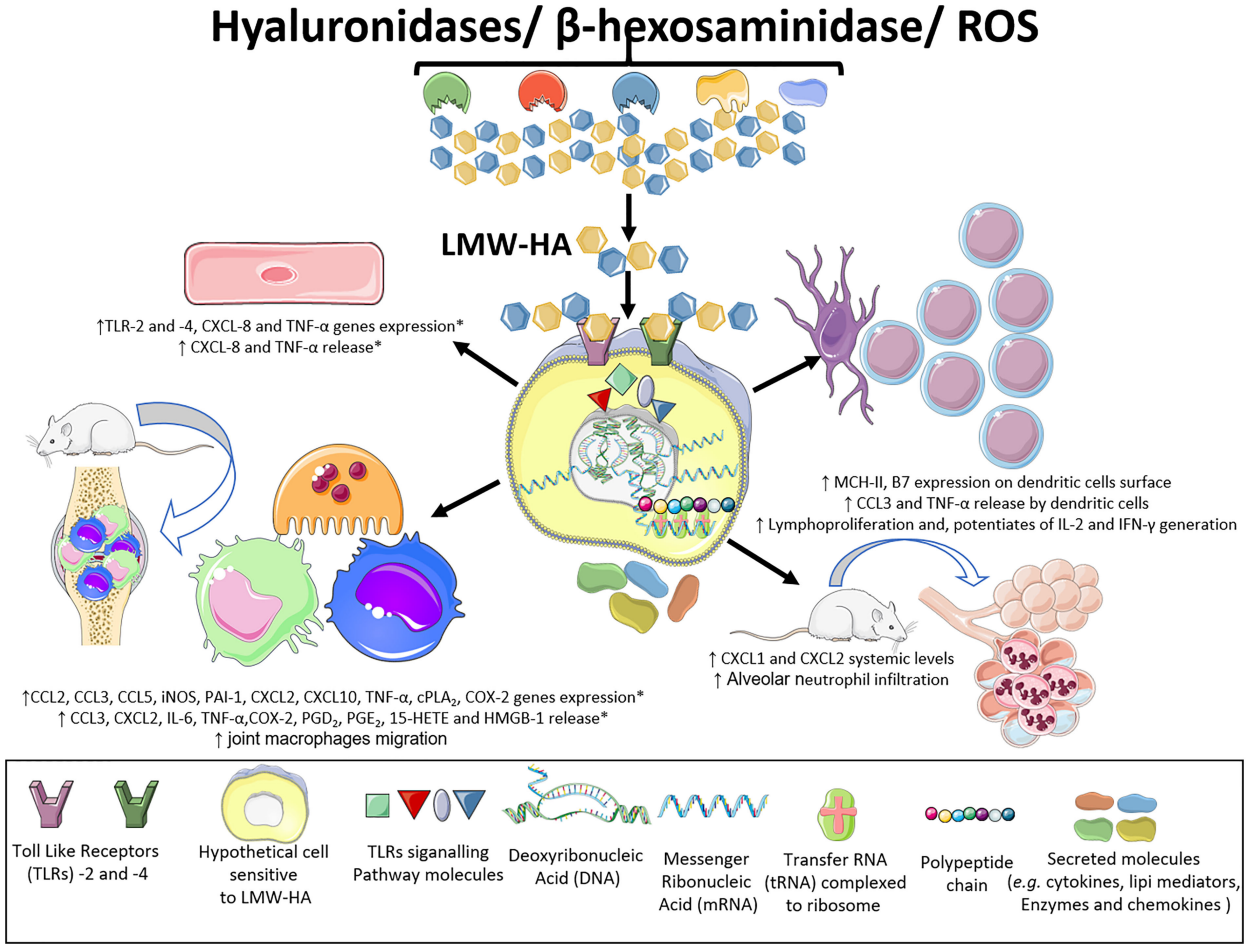
Inflammatory effects of LMW-HA fragments. LMW-HA can be sensed by different cell types. By performing *in vivo, in vitro* and *ex-vivo* experiments, using diverse pharmacological strategies, genetic interference and knock out mice, various studies have shown that LMW-HA fragments, in their DAMP form, are sensed by TLR-2 and -4, on endothelial cells, macrophages and dendritic cells. This event leads to cell activation, with expression of inflammatory genes, production of chemokines, interleukins and lipid mediators, modulation of the expression of cell surface molecules and lymphoproliferation. Besides, it can promote infiltration of macrophages and neutrophils into the synovial and pulmonary tissues causing joint and alveoli destruction, respectively. (*) effects characterized both in mice and human samples. This Figure was partly generated using Servier Medical Art, provided by Servier, licensed under a Creative Commons Attribution 3.0 unported license. Of note, some icons in this figure were adapted for our necessities.

Several investigations have documented that mouse peritoneal, alveolar, and synovial macrophages are overly sensitive to LMW-HA exposure. These cells present increases in CCL2 (C-C motif chemokine ligand 2), CCL3, CCL5, iNOS (inducible nitric oxide synthase), PAI-1 (plasminogen activation inhibitor 1), elastase, CXCL2 (C-X-C motif chemokine ligand 2), CXCL10, and TNF-α (tumor necrosis factor α) gene expression. Additionally, after exposure to LMW-HA, mouse macrophages release large amounts of the cytokines CCL3, CXCL2, interleukin 6 (IL-6), and TNF-α, as well as HMGB-1 (high mobility group box-1), a DAMP (3, 6, 8, 9) (**Figure 4**).

Human macrophages have also been shown to be highly susceptible to the effects of LMW-HA. In response to these stimuli, human macrophages release large amounts of arachidonic acid through mechanisms depending on cPLA2α (cytosolic phospholipase A2), ERK1/2, p38 (p38 MAPK) and JNK (c-Jun N-terminal kinase) phosphorylation. In addition, these cells express of the cyclooxygenase 2 enzyme (COX-2) at high levels and generate high levels of PGE_2_ (prostaglandin E2). LMW-HA, by promoting an increase in inflammation-related gene expression, polarizes macrophages toward the M1 phenotype, making them highly inflammatory. In addition, LMW-HA changes the M2a phenotype (by inducing increased production of PGE_2_, PGD_2_, and 15-HETE) to the M1 phenotype that is characterized as cPLA2αhigh COX-2high and COX1low/ALOX5low/ALOX15low/LTA4Hlow (10) (**Figure 4**).

ECs are another cell type that is influenced by LMW-HA generated by HYAL, including increases in the levels of the chemokine CXCL8 (7) and TNF-α (8), and in TLR-2 and -4 receptor mRNA expression. In addition, human EC activation by HA fragments leads to the release of elevated levels of CXCL8 and TNF-α into the extracellular space (7, 8) (**Figure 4**).

Strikingly, HA fragments also influence several aspects of DC biology. LMW-HA elicits functional maturation of human and mouse DCs, as detected by the increase of MHC-II and B7 molecule expression on the cell surface and TNF-α and CCL3 generation, and by their capacity to promote T cell proliferation *in vitro*. Notably, LMW-HA injection *in vivo* also triggered DC maturation and lymphoproliferation (7) (**Figure 4**).

According to Sheibner and colleagues (9), T cells from OT-II transgenic mice, which express an ovalbumin (OVA)-specific T cell receptor, produce large amounts of interleukin 2 (IL-2) and interferon γ (IFN-γ) when immunized with OVA mixed with LMW-HA fragments. The IL-2 and IFN-γ production in the presence of LMW-HA was increased two and seven times, respectively, compared with animals immunized without these fragments. Thus, LMW-HA fragments appear to trigger systemic reactions *in vivo*. Additionally, these data show that LMW-HA is a potent adjuvant (**Figure 4**).

Intraperitoneal injection of these fragments into mice increases circulating levels of the CXCL1 and CXCL2 chemokines (7), which are homologous to human CXCL8, a potent bone marrow neutrophil mobilizer (49). These inflammatory events also indicate the systemic reactions elicited by HYAL actions upon HA (**Figure 4**).

Although SVHYA are broadly produced by venomous snakes, the immunopathological effects of these enzymes have not been explored. Strikingly, by coupling biological, transcriptomic and bioinformatics approaches, our group identified an outstanding role for HA fragments in a pararamosis cell model. This clinical condition is characterized by osteoarthritis after accidental chronic exposure to the hairs of the larval stage of the Brazilian mouth *Premolis semirufa*, popularly called pararama. Human chondrocyte exposure to the pararama hair extract enhanced the activities of the HA/CD44/TLR-2 and TLR-4/NFκB/AP-1/interleukin/prostanoids/C-C and C-X-C chemokine/collagen III/growth factor/matrix metalloproteinase axes, which are upregulated in individuals with joint disorders (50).

**Figure 5:**
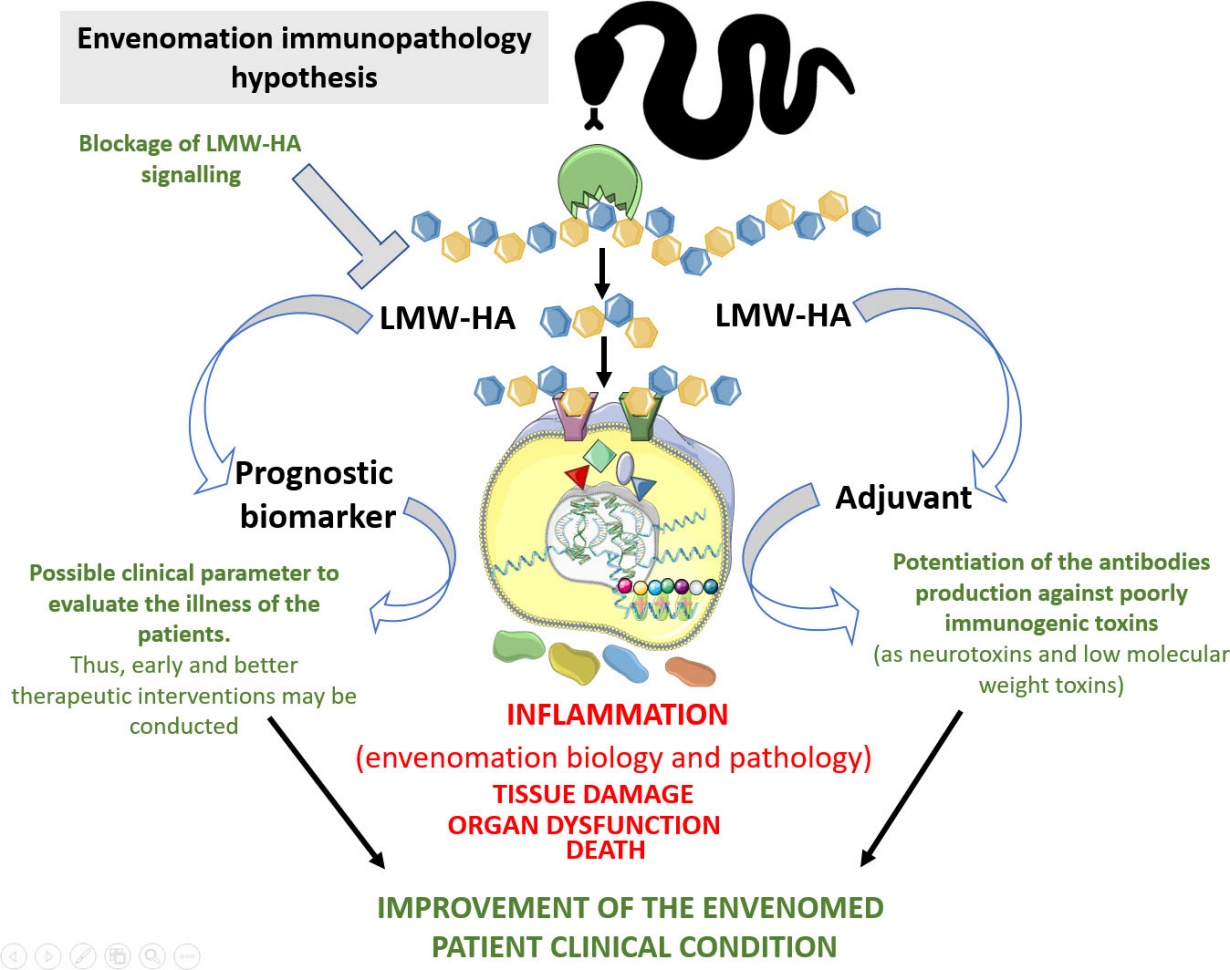
Hypothesis of the immunopathological contributions of LMW-HA to snakebite envenoming. Snake venom hyaluronidases, as well those produced by mammalians, can hydrolyse hyaluronan and release tetra- and hexasaccharides, which in turn will be sensed by TLRs in immune and non-immune cells, thus promoting inflammation. These inflammatory reactions elicited by these fragments will influence biological and pathological aspects of the envenomation by snakes. In the biological side, hyaluronan fragmentation by snake venom hyaluronidases triggers lipid mediators generation. This event can impair the endothelium physical barrier function, which allows the access of venom toxins to the bloodstream and to the cell/tissue targets. This can also promote systemic vasodilation, thus contributing to the prey death. In the pathological scenario, LMW-HA can trigger uncontrolled inflammatory reactions at local and systemic levels, which in turn would promote tissue damage and organ dysfunction, thus contributing to envenomed victim death. On the other hand, the detection of LMW-HA in the blood of the snake bitten patients could serve as parameter for the clinical evaluation of the severity of the envenoming. This would contribute to early and better therapeutic interventions. Additionally, these molecules could be inhibited to mitigate their toxic action in the envenomations. This Figure was partly generated using Servier Medical Art, provided by Servier, licensed under a Creative Commons Attribution 3.0 unported license. Of note, some icons in this figure were adapted for our necessities.

After considering the exposed data, we hypothesize that SVHYA acts on HA in the ECM and bloodstream, triggering several inflammatory events that account for snake envenomation immunopathology (**Figure 5**). By performing *in vivo* and *ex vivo* approaches using crude venom from different venomous snake species in which SVHYA were detected (5, 51, 52), different authors observed several inflammatory events suggestive of the actions of SVHYA enzymes, such as COX-2 enzyme expression (53); CXCL1, CXCL8, TNF-α, IL-6 and PGE_2_ production; acute lung injury; and neutrophil tissue infiltration (5, 54, 55). The production of some inflammatory mediators, such as PGE_2_, *via* the SVHYA/LMW-HA axis could be responsible for pain (56) and systemic vasodilation (57, 58) in envenomed individuals, which could evolve to severe hypotension resulting in circulatory shock and extensive edema.

Surprisingly, LMW-HA fragments are presented as potent adjuvant molecules since potentiates immune response to some antigens, thus these molecules could be used in the antivenom production to increase immunogenicity of some potent neurotoxins/low molecular weight toxins that antibody production is problematic due to low immunogenicity.

Additionally, some inflammatory events elicited directly or indirectly by crude snake venoms might result in HA degradation and, consequently, LMW-HA formation, for example, by the β-hexosaminidase enzyme released from human and mouse mast cells exposed to snake venoms (59) and local and systemic ROS generation after mouse experimental envenomation (60).

Importantly, patients suffering from several clinical illnesses, such as sepsis, acute lung injury, asthma and kidney failure, present increased systemic levels of LMW-HA (10). Notably, elevated levels of LMW-HA in septic patients are correlated with disease severity and a poor prognosis (6, 8). Thus, because envenomated patients present some clinical symptoms similar to those observed in patients with the clinical conditions mentioned above and HYAs are ubiquitously expressed in snake venoms, systemic LMW-HA levels may be used as a biomarker of envenomation severity.

Interestingly, the blockade of LMW-HA actions with a Pep-1 inhibitor abrogated the massive macrophage migration to arthritic joints (8), as well as neutrophil infiltration in alveolar spaces after bleomycin-induced injury (6), which were linked to a decrease in chemokine production. Considering that the production of chemokines and other inflammatory mediators that are present during snakebite envenomation may be partially mediated by LMW-HA, this inhibitory approach may have therapeutic potential to control this clinical condition (**Figure 5**).

In addition to determining the inflammatory properties of envenomation, LMW-HA fragments generated by SVHYA or HYAL might be an interesting adjuvant to improve antivenom production for poorly immunogenic snake venoms, since these fragments might potentiate an adaptive immune response to venom toxins (**Figure 5**).

Additionally, regarding the immunopathological effects, studies investigating whether the canonical function of SVHYA, *e.g.*, toxin delivery to the bloodstream, is influenced by inflammatory mediators will be interesting. Although the dissemination of toxins mediated by the SVHYA in some venoms are assisted by toxins that kill endothelial cells, some venoms do not contain cytotoxins. Thus, toxin delivery mediated by these enzymes may occur *via* endothelial dysfunction caused, for example, by lipid mediators elicited by LMW-HA.

## 4 Concluding remarks

HYAs have been detected in a plethora of animal venoms, which suggests important pathological roles similar to those of other venom components. In recent years, several authors have described diverse molecular mechanisms underlying the deleterious inflammatory reactions to envenomations by snakes (61, 62), caterpillars (50) and scorpions (63–65), making them an immunopathological signature in these accidents. The World Health Organization (WHO) (66) has established actions to decrease snakebite envenoming-associated mortality and disabilities by 50% by 2030. This program includes incentives for studies of “next-generation” therapies, whose development will require the best characterization of the molecular mechanisms involved in envenomation pathology. Thus, the inflammatory actions of animal venom hyaluronidases may provide new opportunities for the development of novel therapeutic targets prognostic biomarker for snakebite envenoming. Additionally, the use of HA-LMW as an adjuvant to increase B and T cell responses and improve the humoral response to poorly immunogenic toxins in antivenom-producing animals should be considered.

## Author contributions

FSF and DVT conceived the study. FSF drew all the figures. FSF and DVT wrote the manuscript. All authors contributed to the article and approved the submitted version.
